# Fellow-Workers and the Roll of Honour

**Published:** 1914-12-05

**Authors:** 


### ROUND THE WORLD.

[The Editor Invites Early Information and Contributions Marked "Fellow-Workers."]

### Fellow-Workers and the Roll of Honour.

Mr. George Robertson Turner, F.R.C.S. Eng., one of the surgeons appointed as consultant at Chatham, is surgeon and joint lecturer on surgery at St. George's Hospital, to which he has been attached for many years. He is also consulting surgeon to the Railway Passengers' Assurance Company and holds the rank of major in the Territorial branch of the R.A.M.C. Mr. Turner was for eighteen years visiting surgeon to the Seamen's (*Dreadnought*) Hospital at Greenwich.

Mr. Cecil Hugh Myddelton Hughes, M.B., B.S. Lond., who has been attached to the Royal Naval Hospital at Chatham in a similar capacity, served with the South African Field Force in 1900, and wears a medal and three clasps as a result of his experiences then. A specialist in anæsthetics, he holds the posts of assistant anæsthetist to the Westminster Hospital, where he was formerly a student, and anæsthetist to the Paddington Green Children's Hospital. His past appointments include those of anæsthetist and lecturer in anæsthetics to the Seamen's (*Dreadnought*) Hospital, Greenwich, and anæsthetist to the National Dental Hospital.

The Admiralty have also appointed Dr. H. D. Rolleston to be temporary consulting physician, together with Mr. G. Lenthal Cheatle and Mr. C. W. Morris as temporary consulting surgeons, to the Naval Hospital at Haslar.

Dr. Humphry Davy Rolleston is a St. Bartholomew's man, and was elected a member of the staff of St. George's Hospital, where he is now senior physician, many years ago. A well-known authority on diseases of abdominal organs, Dr. Rolleston is also known to a wide circle of medical men as joint editor of the second edition of "Allbutt's System of Medicine." Qualifying M.B., B.C. Cantab. in 1888, and becoming a member of the Royal College of Physicians in the following year, this distinguished consultant was elected to the dignity of the fellowship thereof only five years later. Amongst other appointments held by Dr. Rolleston may be mentioned those of physician to the Victoria Hospital for Children, consulting physician to the West Herts Hospital, and examiner in medicine to the universities of London, Oxford, Cambridge, and Manchester, as well as to the Naval and Army Medical Boards.

Mr. George Lenthal Cheatle, C.V.O., C.B., is surgeon and lecturer on operative surgery to King's College Hospital, and consulting surgeon to the Hospital for Paralysis and Epilepsy in Maida Vale. A student of King's College, he became an F.R.C.S. Eng. in 1890, and rapidly made a name for himself as an operative surgeon. At the time of the Boer War, Mr. Cheatle went to South Africa as consulting surgeon to His Majesty's Forces, being mentioned in despatches, and coming home with a medal and four clasps and a C.B. Mr. Cheatle was for many years surgeon to the Italian Hospital, London, and was made an officer of the Grand Cross of Italy when a member of the Royal Italian family visited the institution four or five years ago.

Mr. Raymond Johnson, who is temporary consulting surgeon to the Naval Hospital at Plymouth, is a prominent member of the staff of University College Hospital, and an examiner in surgery to the Universities of Cambridge and London. He won the University scholarship in forensic medicine, together with the gold medal in obstetric medicine, with honours in medicine, at the M.B. Lond. examination in 1886. Taking the B.S. in the same year he became an F.R.C.S. Eng. two years later. At the time of the South African War Mr. Raymond Johnson, who was for many years surgeon to the Great Northern Central Hospital in Holloway, went to the Front as surgeon to the Imperial Yeomanry Hospital. He is the author of a volume of University College Hospital surgical reports, and the joint editor of the tenth edition of Erichsen's well-known text-book of surgery.

Sir Alfred Downing Fripp, K.C.V.O., C.B., who, with Dr. D. A. Shields, has been appointed by the Admiralty as temporary consultant to His Majesty's hospital ship *Sheelah*, is, of course, a well-known leading West End surgeon. Qualifying from Guy's Hospital he eventually became an M.S. Lond. and F.R.C.S., whilst the excellence of his early work secured him election to the staff of his hospital, where he is now one of the senior surgeons and lecturer on surgery. Sir Alfred Fripp went to South Africa during the war as senior surgeon and chief civilian medical officer to the Imperial Yeomanry Hospital, and published some interesting letters on his experiences in the medical Press at that time. Formerly surgeon-in-ordinary to King Edward VII., Sir Alfred Fripp holds a similar appointment in the establishment of His Majesty the King, and is also surgeon-in-ordinary to His Royal Highness the Duke of Connaught. A Knight of Grace of the Order of St. John of Jerusalem in England, he received a C.B. in 1900, was knighted three years later, and became a K.C.V.O. in 1906.

Dr. Douglas Andrew Shields, who has likewise been appointed to His Majesty's ship *Sheelah*, also practises in the West End. Educated at Melbourne University, he qualified M.B., Ch.B. Melbourne in 1897 with first-class honours, and became an M.D. of his university four years later. He was at one time surgeon to his Excellency the Governor-General of Australia, senior surgeon to the St. Vincent's Hospital, Melbourne, and lecturer on clinical surgery in the university there.

Sir William Macewen, whose services have also been secured by the Admiralty, is one of the honorary surgeons to His Majesty the King in Scotland, Regius Professor of surgery in Glasgow University, and probably the best known consulting surgeon north of the Border. An F.R.S., the recipient of various honorary degrees, and honorary member of various foreign academies and societies, Sir William Macewen is particularly famous on account of his researches and methods of treatment of diseases of the central nervous system and bones. He was knighted in 1902.

